# Cardioprotective effects of Moku-boi-to and its impact on AngII-induced cardiomyocyte hypertrophy

**DOI:** 10.3389/fcell.2023.1264076

**Published:** 2023-11-07

**Authors:** Hideaki Tagashira, Fumiha Abe, Kaori Sato-Numata, Karen Aizawa, Kei Hirasawa, Yoshinobu Kure, Daiki Iwata, Tomohiro Numata

**Affiliations:** ^1^ Department of Integrative Physiology, Graduate School of Medicine, Akita University, Akita, Japan; ^2^ School of Medicine, Akita University, Akita, Japan

**Keywords:** Kampo medicine, cardiomyocyte hypertrophy, mitochondria, angiotensin-receptor blocker, mitochondria fission/fusion dynamics, ROS, Ca^2+^ homeostasis, cell volume regulation

## Abstract

Cardiomyocyte hypertrophy, induced by elevated levels of angiotensin II (AngII), plays a crucial role in cardiovascular diseases. Current therapeutic approaches aim to regress cardiac hypertrophy but have limited efficacy. Widely used Japanese Kampo medicines are highly safe and potential therapeutic agents. This study aims to explore the impact and mechanisms by which Moku-boi-to (MBT), a Japanese Kampo medicine, exerts its potential cardioprotective benefits against AngII-induced cardiomyocyte hypertrophy, bridging the knowledge gap and contributing to the development of novel therapeutic strategies. By evaluating the effects of six Japanese Kampo medicines with known cardiovascular efficiency on AngII-induced cardiomyocyte hypertrophy and cell death, we identified MBT as a promising candidate. MBT exhibited preventive effects against AngII-induced cardiomyocyte hypertrophy, cell death and demonstrated improvements in intracellular Ca^2+^ signaling regulation, ROS production, and mitochondrial function. Unexpectedly, experiments combining MBT with the AT_1_ receptor antagonist losartan suggested that MBT may target the AT_1_ receptor. In an isoproterenol-induced heart failure mouse model, MBT treatment demonstrated significant effects on cardiac function and hypertrophy. These findings highlight the cardioprotective potential of MBT through AT_1_ receptor-mediated mechanisms, offering valuable insights into its efficacy in alleviating AngII-induced dysfunction in cardiomyocytes. The study suggests that MBT holds promise as a safe and effective prophylactic agent for cardiac hypertrophy, providing a deeper understanding of its mechanisms for cardioprotection against AngII-induced dysfunction.

## Introduction

Heart Failure (HF) occurs when the heart cannot pump enough blood to meet the body’s needs, causing various symptoms such as shortness of breath, fatigue, and swelling of the legs and ankles. It is a rapidly growing public health problem, affecting at least 20–40 million people worldwide (Ziaeian and Fonarow, 2016; Savarese and Lund, 2017). Drug therapy is a cornerstone of HF treatment to address this problem. Various drugs targeting G protein-coupled receptors and ion channels are available to prevent the deterioration of ventricular systolic function and reduce mortality in patients (Rahm et al., 2018; Thai et al., 2023). On the other hand, medications used for conditions other than HF, such as antihypertensives and antidiabetic drugs, are prescribed only to people with pre-existing conditions because they can make HF worse (Page et al., 2016). Therefore, there is an urgent need to develop more effective therapeutic agents to improve the quality of life of patients and reduce the burden of medical costs associated with HF.

Excessive activation of a bioactive substance, angiotensin II (AngII), has been suggested to be involved in the onset and progression of HF. AngII induces physiological changes such as vasoconstriction, fibrosis, and cell death, associated with impaired mitochondrial function (Benigni et al., 2010). Recent studies have shown that continuous intracellular Ca^2+^ signaling is the cause of cardiac hypertrophy and HF (Bers, 2006). Furthermore, intracellular Ca^2+^ homeostasis regulation has been shown that a good balance between mitochondrial fission and fusion is critical for maintaining mitochondrial morphology and function in the heart and that disruption of this balance leads to the development of heart disease (Brown et al., 2017; Zhou and Tian, 2018; Yang et al., 2021). Therefore, excessive stimulation of AngII to cardiomyocytes that exacerbates HF can lead to abnormally elevated intracellular Ca^2+^ concentration [(Ca^2+^)_i_] levels, leading to cell death associated with mitochondrial dysfunction and cell volume regulation failure (Hunyady and Catt, 2006; Okada et al., 2019).

Moku-boi-to (MBT), a Japanese herbal medicine, is composed of four crude drugs: Sekkou; *Gypsum Fibrosum*, Keishi; bark of *Cinnamomumi* cassia Blume, Boui; *Sinomeni Caulis et Wilson*, and Ninjin; roots of *Panax ginseng C.A. Mayer*. MBT has an antiarrhythmic effect on the myocardium, and sinomenine, a crude drug component contained in MBT, exerts a myocardial protective effect by reducing Ca^2+^ influx into cells (Satoh, 2005; Satoh, 2017). It is empirically known to be effective mainly for renal edema and HF and has been used clinically in Japan for centuries (Yaku et al., 2022).

In this study, we used an AngII-induced cardiomyocyte hypertrophy model to evaluate the effects of MBT on AngII-induced mitochondrial dysfunction and cell death. Furthermore, we investigated the regulatory mechanisms of calcium homeostasis and elucidated the impact of MBT on this process.

## Materials and methods

### Materials

The following Japanese Kampo medicines were used in this study: Sho-seiryu-to (TJ-19), Boi-ogi-to (TJ-20), Mao-to (TJ-27), Moku-boi-to (MBT) (TJ-36), Yokukan-san (TJ-54), and Mashinin-gan (TJ-126). These Japanese Kampo medicines were obtained from TSUMURA & CO. (Tokyo, Japan). Each reagent was dissolved in dimethyl sulfoxide (DMSO) at a concentration of 250 mg/mL and diluted to the desired final concentration as aliquots.

Isoproterenol hydrochloride (ISO) was purchased from Sigma-Aldrich (MO, United States). Losartan potassium and carboxymethyl cellulose sodium salt (CMC) was purchased from Fujifilm Wako (Osaka, Japan). Angiotensin II (AngII) was purchased from Peptide Institute Inc. (Osaka, Japan). Immunostaining reagents, including rhodamine-conjugated phalloidin were obtained from Abcam (ab235138, Abcam, Cambridge, United Kingdom), and MitoTracker Red CMXRos were obtained from Invitrogen (CA, United States). The reactive oxygen species (ROS) indicator, H_2_DCFDA, was obtained from Invitrogen, and the Ca^2+^ indicator, Fluo-4, was obtained from Dojindo (Kumamoto, Japan).

### Animals

All animal-related procedures were conducted in strict accordance with the Guide for Care and Use of Laboratory Animals and received prior approval from the Animal Ethics Committee of Akita University (Akita, Japan). The ethics review number for these protocols were a-1-0412 and b-1-0408. For neonatal rat ventricular myocytes (NRVMs), to obtain neonatal rat ventricular myocytes (NRVMs) for the experiment, Wistar rats aged 1–3 days after birth were used. For the mice experiments, 10–12 weeks old male C57BL/6J mice, bred to the local environment, were employed. These mice were housed in polypropylene cages under controlled environmental conditions, which incubated a temperature of 25°C ± 1°C, humidity regulation, and a 12-h light/dark cycle.

### ISO injection and drug administration

ISO group mice were treated with daily intraperitoneal injections of ISO (30 mg/kg body weight, once a day) (Sigma-Aldrich), following the established protocol detailed in previous studies (Park et al., 2018; Cheng et al., 2020). MBT was dissolved in 0.5% CMC (Fujifilm Wako). Vehicle and MBT (500 mg/body weight, once a day, p.o.) was administered orally for 14 days (once daily) in a volume of 1 mL/100 g of a body weight of mice, starting from the onset of ISO injection.

### Echocardiography and measurement of cardiac hypertrophy

Noninvasive echocardiographic measurements were conducted on anesthetized mice using a mixture of ketamine (50 mg/kg body weight, i.p.) (Daiichi Sankyo Pharmaceutical Co., Ltd., Tokyo, Japan) and xylazine (5 mg/kg body weight, i.p.) (Bayer Yakuhin, Ltd., Osaka, Japan). A VisualSonics Vevo 770 system, equipped with a 30-MHz linear transducer, was used (FUJIFILM VisualSonics Inc., Toronto, Canada). To visualize the heart, two-dimensional parasternal short-axis views were obtained, and an M-mode echocardiogram of the midventricle was recorded, focusing on the papillary muscles. Echocardiographic measurements included diastolic and systolic LV wall thickness, LV end-diastolic diameter (LVEDD), and LV end-systolic diameter (LVESD). All measurements adhered to the guidelines the American Society of Echocardiography set forth and were taken from leading edge to leading edge. The percentage of LV fractional shortening (FS%) was computed using the formula [(LVEDD—LVESD)/LVEDD] × 100. Two-dimensional (2D)-guided M-mode measurements were used to determine both FS% and the percentage of LV Ejection Fraction (EF%). Following the completion of echocardiography and before sacrifice, mice were euthanized through cervical spine fracture-dislocation for cardiac hypertrophy assessment. Subsequently, the thoracic cavity was opened, and the hearts were promptly excised and weighed.

### Mouse tissue collection, histochemical staining

For mouse tissue collection, fixation, and sectioning, the mice were initially weighed and then subjected to deep anesthesia through a mixture of ketamine (100 mg/kg body weight, i.p.) (Daiichi Sankyo Pharmaceutical Co., Ltd.) and xylazine (10 mg/kg body weight, i.p.) (Bayer Yakuhin, Ltd.). Subsequently, their hearts were removed, any blood was carefully wiped away, and the heart weights were measured. The hearts were then fixed in 4% paraformaldehyde phosphate buffer solution (Nacalai Tesque, Kyoto, Japan) at 4°C overnight and subsequently embedded in paraffin. Tissue sections with a thickness of 3 μm were obtained from various locations. For histochemical staining, the tissue sections were deparaffinized and subjected to H&E (HE: hematoxylin and eosin) and Masson’s trichrome (MT) staining following standard protocols. The slides were observed and imaged using a BZ-X800 Keyence inverted microscope (Osaka, Japan). Heart sections from a minimum of 5 mice per group were analyzed. To account for individual variations, the heart weight presented in Figure 7G was calculated by dividing the heart weight by the body weight and expressed as heart weight (HW)/body weight (BW) (mg/g).

### Cell culture

NRVMs were isolated from the hearts of 1-3-day-old Wistar rats. The rat pups were sacrificed by decapitation. NRVMs were isolated using the Pierce cardiomyocyte isolation kit (Thermo Fisher Scientific, MA, United States) following the provided protocol. Briefly, the heart tissue was minced using scissors and subjected to enzymatic treatment. The tissue was then filtered through a 100 μm filter to remove large tissue fragments. The enzyme in the medium was removed by washing it with Hank’s Balanced Salt Solution (HBSS) buffer (Thermo Fisher Scientific). The cell suspension was subsequently resuspended in Dulbecco’s Modified Eagle Medium (DMEM) (Thermo Fisher Scientific) and gently agitated by pipetting. The NRVMs were seeded onto uncoated 100 mm culture dishes and placed in a CO_2_ incubator at 37°C for 90 min; this incubation period allowed non-cardiomyocytes that did not attach to the bottom of the culture dish to be removed. After the incubation, the NRVMs adhering to the culture dish were recovered. The number of NRVMs was determined based on the specific requirements of each experiment. The cells were cultured for 3–4 days before the experiments were conducted. In accordance with the manufacturer’s manual and consistent with previous findings (Ali et al., 2019), our cell population exhibited high cardiomyocyte purity, with over 95% of cells testing positive for cardiac troponin T (cTnT) (cTnT-positive cells: 95.8% ± 2.6%, *n* = 5), while endothelial cells {platelet endothelial cell adhesion molecule-1 [PECAM-1 (CD31)]-positive cells} were minimally present (PECAM-1-positive cells: 3.8% ± 2.3%, *n* = 5). We used cTnT antibody (1:500) (sc-20025, Santa Cruz, CA, United States) and PECAM-1 antibody (1:500) (sc-376764, Santa Cruz) for immunostaining, and confirmation was obtained through fluorescence microscopy (BZ-X800, Keyence).

### Morphological analysis and immunocytochemistry of NRVMs

Morphological analysis of NRVM hypertrophy and immunocytochemistry were conducted following previously described methods (Tagashira et al., 2023). NRVM cells subjected to each experimental condition were fixed with 4% formaldehyde. The fixed cells were then blocked by incubation with 1% bovine serum albumin (BSA) in phosphate-buffered saline (PBS). For cell size measurements, the fixed cells were incubated with rhodamine-phalloidin reagent (Abcam) in PBS containing 1% BSA for 1 h at room temperature. Mitochondrial staining was performed as previously described (Tagashira et al., 2023). For mitochondrial membrane potential and size measurements, the cells were stained with 0.1 μM MitoTracker Red CMXRos (M7512, Invitrogen) in serum-free DMEM for 30 min. For immunocytochemistry, the cells were incubated with anti-dynamin related protein 1 (DRP1) antibody (sc-101270, Santa Cruz) (1:200) in PBS containing 1% BSA at 4°C for overnight to assess DRP1 morphology.

Random cell images were captured using a fluorescence microscope (BZ-X800, Keyence, Osaka, Japan) with a BZ-X filter for cell size measurements. Cross sectional area (CSA) was quantified using the ImageJ program (Schneider et al., 2012). All cells from randomly selected fields were analyzed, and a minimum of 100 myocytes were measured for each experimental condition. Immunofluorescence images for DRP1, mitochondrial membrane potential, and mitochondrial morphology measurements were acquired randomly using a confocal laser scanning microscope (LSM980, Carl Zeiss Microscopy, Jena, Germany) at the appropriate excitation/emission wavelengths. Fluorescence intensity and mitochondrial length were measured using the ImageJ program. For mitochondrial length analysis, at least 60 mitochondria were calculated for each experimental condition, including all mitochondria within the cells (Jong et al., 2019). The Calmpfit software (Clampex 11.2, Molecular Devices, CA, United States) was utilized to determine the average value obtained through Gaussian fitting of the distribution map, with the length of mitochondria plotted along the horizontal axis. The 50% inhibition concentration (IC_50_) of MBT was calculated in GraphPad Prism 9 (GraphPad software, CA, United States), using the sigmoidal dose-response equation “log [inhibitor] vs. response–variable slope,” defined as:

$$Y=\text{Bottom}+\left(\text{Top}-\text{Bootom}\right)/\left({1+10}^{{\left(\text{LogIC}50-X\right)}^{*}\text{Hill slope}}\right)$$

Where “Top” represents the maximal response, “Bottom” represents the maximally inhibited response, and “Hill slope” represents the steepness of the curve.

### Measurement of cell viability and cytotoxicity

Cell viability was assessed using a colorimetric MTT assay kit (Cell counting kit-8; Dojindo). NRVMs were cultured at 4.0 × 10^5^ cells/mL in 96-well plates and maintained in DMEM. Measurements were conducted 72 h after treatment with 100 nM AngII using absorbance at 450 nm wavelengths on a Multiskan JX plate reader (model 353, Thermo Fisher Scientific). The cell viability depicted in Figures 2E, 5C is presented as a relative value (%) of the absorbance obtained in each experiment, normalized to the average value of the control condition. To assess cardiomyocyte cytotoxicity, we employed a colorimetric lactate dehydrogenase (LDH) activity assay kit (Cytotoxicity LDH Assay Kit-WST, Dojindo) following the manufacturer’s protocol. NRVMs were plated at a 4.0 × 10^5^ cells/mL density in 96-well plates and cultured in DMEM. Measurements were conducted 72 h after exposure to 100 nM AngII. The release of LDH activity was quantified using an Infinite M200 microplate reader (Tecan Group Ltd., Männedorf, Switzerland) by measuring the absorbance of the formazan dye at an optical wavelength of 490 nm. Cytotoxicity data, as presented in Figures 2F, 5D, were calculated in accordance with the manufacturer’s instructions.

### Measurement of intracellular Ca^2+^, ROS, and ATP content

[Ca^2+^]_i_ measurements using Fluo-4 AM (F312, Dojindo) and ROS measurements using H_2_DCFDA (C400, Invitrogen) were conducted following the manufacturer’s protocol. NRVMs were cultured in 96-well plates at 4.0 × 10^5^ cells/mL and maintained in DMEM. [Ca^2+^]_i_ and ROS measurements were performed 48 h after treatment with 100 nM AngII. For [Ca^2+^]_i_ measurements, cells were incubated with 5 μM Fluo-4 AM in Hank’s Balanced Salt Solution (HBSS) (Thermo Fisher Scientific) containing 1 mM CaCl_2_ for 30 min at 37°C in a CO_2_ incubator. After washing with HBSS, [Ca^2+^]_i_ levels at excitation/emission wavelength = 485/538 nm were quantified using a fluorescence microplate reader (Fluoroskan Ascent, Thermo Fisher Scientific). For ROS measurements, cells were treated with 5 μM H_2_DCFDA in HBSS for 30 min at 37°C in a CO_2_ incubator. Subsequently, the cells were washed with HBSS, and ROS levels at excitation/emission wavelength = 485/538 nm were measured using a fluorescence microplate reader (Fluoroskan Ascent, Thermo Fisher Scientific). ATP measurement was performed using an ATP assay kit (Fujifilm Wako), following the manufacturer’s protocol as previously described (Tagashira et al., 2023). Luminescence was recorded using a Lumitester C-110 (Kikkoman Biochemifa, Tokyo, Japan). The ATP content presented in Figure 2G is expressed as a relative value (%) of the absorbance obtained in each experiment, normalized to the average value of the control condition.

### RNA isolation and real time-PCR

RNA isolation followed a previously described protocol (Numata et al., 2021). Total RNA was extracted from NRVMs using the NucleoSpin^®^ RNA Plus kit (Takara-Bio, Otsu, Japan) according to the manufacturer’s instructions. The concentration and purity of the extracted RNA were assessed using a Nanodrop-ND1000 spectrophotometer (Thermo Fisher Scientific). Reverse transcription of the total RNA samples was performed using the Prime-Script™ II 1st Strand cDNA Synthesis Kit (Takara-Bio) with Prime-Script RTase for 60 min at 42°C, following the manufacturer’s protocol. Quantitative real-time PCR was carried out using the SYBR Green Real time PCR Master Mix -Plus (Toyobo Co., Ltd., Osaka, Japan) on a LightCycler^®^ 480 real-time PCR system (Roche Diagnostics Ltd., Rotkreuz, Switzerland), following the manufacturer’s instructions. Gene-specific primer sequences were synthesized by FASMAC (Kanagawa, Japan). The rat primer sequences used were as follows: Atrial natriuretic peptide (ANP) (NM_012612.2, 105 bp) forward and reverse primers: 5′-AAA​TCC​CGT​ATA​CAG​TGC​GG-3′, and 5′-GGA​GGC​ATG​ACC​TCA​TCT​TC-3′, respectively (Böckmann et al., 2019); Brain natriuretic peptide (BNP) (NM_031545.1, 364 bp) forward and reverse primers: 5′-CCA​TCG​CAG​CTG​CCT​GGC​CCA​TCA​CTT​CTG-3′, and 5′-GAC​TGC​GCC​GAT​CCG​GTC-3′, respectively (Birnbaum et al., 2019); β-myosin heavy chain (β-MHC) (NM_017240.2, 238 bp) forward and reverse primers: 5′-TGC​AGT​TAA​AGG​TGA​AGG​C-3′, and 5′-CAG​GGC​TTC​ACA​GGC​AT-3′, respectively (Liu et al., 2018); Regulator of calcineurin 1 (RCAN1) (NM_153724.2, 206 bp) forward and reverse primers: 5′-GCC​CGT​TGA​AAA​AGC​AGA​AT-3′, and 5′-GAC​AGG​GGG​TTG​CTG​AAG​TT-3′, respectively (Friedrich et al., 2014); AT_1A_R (NM_030985.4, 306 bp) forward and reverse primers: 5′-CGT​CAT​CCA​TGA​CTG​TAA​AAT​TTC-3′, and 5′-GGG​CAT​TAC​ATT​GCC​AGT​GTG-3′, respectively (Miyata et al., 1999); AT_1B_R (NM_031009.2, 345 bp) forward and reverse primers: 5′-CAT​TAT​CCG​TGA​CTG​TGA​AAT​TG-3′, and 5′-GCT​GCT​TAG​CCC​AAA​TGG​TCC-3′, respectively (Miyata et al., 1999); AT_2_R (NM_012494.4, 1126 bp) forward and reverse primers: 5′- TTG​CTG​CCA​CCA​GCA​GAA​AC-3′, and 5′-GTG​TGG​GCC​TCC​AAA​CCA​TTG​CTA-3′, respectively (Xu et al., 2002); Sodium-calcium exchanger 1 (NCX1) (NM_019268.4, 334 bp) forward and reverse primers: 5′-GGA​TGT​GGT​TGA​AAA​TGA​CCC​AGT-3′, and 5′-TAT​GCC​ATC​TTC​CGA​GAC​TTC​TGA-3′, respectively (Nagano et al., 2004); Ryanodine receptor 1 (RyR1), (NM_001419537.1, 101 bp) forward and reverse primers: 5′-CAA​GCG​GAA​GGT​TCT​GGA​CA-3′, and 5′-TGT​GGG​CTG​TGA​TCT​CCA​GAG-3′, respectively (Ferretti et al., 2015); RyR2 (NM_032078.3, 130 bp) forward and reverse primers: 5′-CAT​CGG​TGA​TGA​AAT​TGA​AGA-3′, and 5′-AGC​ATC​AAT​GAT​CAA​ACC​TTG-3′, respectively (Jurkovicova et al., 2007); type 2 inositol 1,4,5-trisphosphate receptor (IP_3_R2) (NM_031046.4, 362 bp) forward and reverse primers: 5′-GCT​CTT​GTC​CCT​GAC​ATT​G-3′, and 5′-CCC​ATG​TCT​CCA​TTC​TCA​TAG​C-3′, respectively (Jurkovicova et al., 2007); DRP1 (NM_053655.3, 148 bp) forward and reverse primers: 5′-AGA​ATA​TTC​AAG​ACA​GCG​TCC​CAA​AG-3′, and 5′-CGC​TGT​GCC​ATG​TCC​TCG​GAT​TC-3′, respectively (da Silva et al., 2014); Translocase of the outer mitochondrial membrane 20 (Tom20) (NM_152935.2, 198 bp) forward and reverse primers: 5′-TGG​GCT​TTC​CAA​GTT​ACC​TGA​TT-3′, and 5′-ACT​GGT​GGT​GGA​AGA​GTC​TGT​TGT​A-3′, respectively (Tian et al., 2022); Voltage-dependent anion channel 1 (VDAC1) (NM_031353.2, 242 bp) forward and reverse primers: 5′-CAA​CAC​GGA​GAC​CAC​CAA​AG-3′, and 5′-CAC​AGC​CCA​GGT​TGA​TAT​GC-3′, respectively (Li et al., 2023); Mitofusin 1 (Mfn1) (NM_138976.2, 140 bp) forward and reverse primers: 5′-CTC​GGA​ATC​AAC​GCT​GAT​GAA​C-3′, and 5′-TGC​GCA​CAT​CCT​CCA​TAT​ATT​CT-3′, respectively (Leduc-Gaudet et al., 2018); Mfn2 (NM_130894.4, 412 bp) forward and reverse primers: 5′-CTC​AGG​AGC​AGC​GGG​TTT​ATT​GTC​T-3′, and 5′-TGT​CGA​GGG​ACC​AGC​ATG​TCT​ATC​T-3′, respectively (Gao et al., 2012); Opa1 (NM_133585.3, 189 bp) forward and reverse primers: 5′-AAG​AAC​CTG​GAA​TCT​CGA​GGA​GTC​G-3′, and 5′-CCA​GAA​CAG​GAC​CAC​GTC​GTT​GC-3′, respectively (da Silva et al., 2014); Mitochondrial fission 1 protein (Fis1) NM_001401051.1, 130 bp) forward and reverse primers: 5′-ACA​ATG​ACG​ACA​TCC​GTA​GAG​G-3′, and 5′-GCC​TTT​TCA​TAT​TCC​TTG​AGC​CG-3′, respectively (Leduc-Gaudet et al., 2018); Mitochondrial fission process protein 1 (Mtfp1) (NM_001006960.1, 102 bp) forward and reverse primers: 5′-AGA​TGA​AGG​CCC​TGA​GGA​GT-3′, and 5′-CCA​GTG​TTC​GTT​CCC​ACT​CA-3′, respectively (designed by authors using Primer-BLAST); β-actin (NM_031144.3, 77 bp) forward and reverse primers: 5′-ACT​ATC​GGC​AAT​GAG​CGG​TTC-3′, and 5′-ATG​CCA​CAG​GAT​TCC​ATA​CCC-3′, respectively (Bao et al., 2017); Glyceraldehyde-3-phosphate dehydrogenase (GAPDH) (NM_017008.4, 140 bp) forward and reverse primers: 5′-GCA​AGT​TCA​ACG​GCA​CAG-3′, and 5′-GCC​AGT​AGA​CTC​CAC​GAC​AT-3′, respectively (Liu et al., 2018). PCR was conducted using KOD-Plus-Ver.2 (Toyobo) with the following conditions: initial denaturation at 94°C for 2 min, followed by 35 cycles of denaturation at 98°C for 10 s, annealing at 55°C for 30 s, and a final extension at 68°C for 60 s. The real-time PCR reactions were performed in triplicate for each sample, and the cycling conditions were as follows: initial denaturation at 95°C for 30 min, followed by 40 cycles of denaturation at 95°C for 5 s, annealing at 55°C for 10 s, and extension at 72°C for 90 s. Melting curve analysis was performed to verify the specificity of the amplification. The relative expression levels of the target genes were normalized to the expression of the housekeeping gene *GAPDH*. To ensure the reliability of the normalization process, we also evaluated the expression of another housekeeping gene, *β-actin*. This analysis was conducted meticulously and confirmed no significant differences compared to the expression levels of *GAPDH*. The data were analyzed using the ΔΔCt method to calculate the mRNA level. Additionally, a representative gel image of the real-time PCR products was captured using gel electrophoresis to confirm the specificity of the amplification. The PCR products were separated on a 2% agarose gel and visualized using GelRed™ (Fujifilm Wako) staining.

### Western blot analysis

Western blot analysis followed established procedures, as previously described (Numata et al., 2021). Briefly, NRVMs were lysed in radio-immunoprecipitation assay (RIPA) buffer (Nacalai Tesque) and centrifuged at 15,000 g for 15 min. Whole-cell lysates were fractionated by SDS-PAGE (sodium dodecyl sulfate-polyacrylamide gel electrophoresis) on either 10% polyacrylamide gels. Following electrophoresis, proteins were transferred onto a polyvinylidene fluoride (PVDF) membrane. The PVDF membrane was then incubated with primary antibodies, specifically anti-DRP1 antibody (1:500) (sc-101270, Santa Cruz) and anti-β-actin antibody (1:2000) (A1978, Sigma-Aldrich) as internal standard. After the primary antibody incubation, the blots were incubated with the secondary antibody of mouse IgG (1:2000) (NA9310, Amersham, Little Chalfont, United Kingdom). Immunoreactivities were visualized using a chemiluminescence reagent (ImmunoStar Zeta, Fujifilm Wako). The chemiluminescence emitted by the membrane was detected using the LumiVision Pro 400EX system (Aisin Seiki Co., Ltd., Aichi, Japan). Quantitative analysis of the Western blot results was performed using the ImageJ program.

### Statistical analysis

Statistical analysis and preparation of figures were performed using GraphPad prism software (version 9, GraphPad software). Results were expressed as mean ± standard error of the mean (SEM). To assess the difference between two means, the Student’s t-test was employed after conducting an F-test to verify the equality of variances within the population of assessments. A *p*-value of *p* < 0.05 was considered statistically significant to determine the presence of a significant difference. Experimental procedures were repeated at least three times independently to ensure reproducibility of results. Data are represented visually using bar or line graphs, mean values are shown, and error bars represent SEM. We followed Chou’s paper as a reference in calculating the combination index (CI) (Chou, 2010).

## Results

### Effect of MBT on AngII-induced cardiomyocyte hypertrophy and death in cultured NRVMs

Angiotensin II (AngII) is a well-established inducer of cardiomyocyte hypertrophy (Sadoshima and Izumo, 1993). Previous studies have commonly used a concentration of 100 nM AngII to induce hypertrophy and cell death in cardiomyocytes (Huang et al., 2014; Li et al., 2014; Chhor et al., 2023). Therefore, we employed primary neonatal rat ventricular cardiomyocytes (NRVMs) and induced cardiomyocyte hypertrophy by treating them with 100 nM AngII in this study.

**FIGURE 1:**
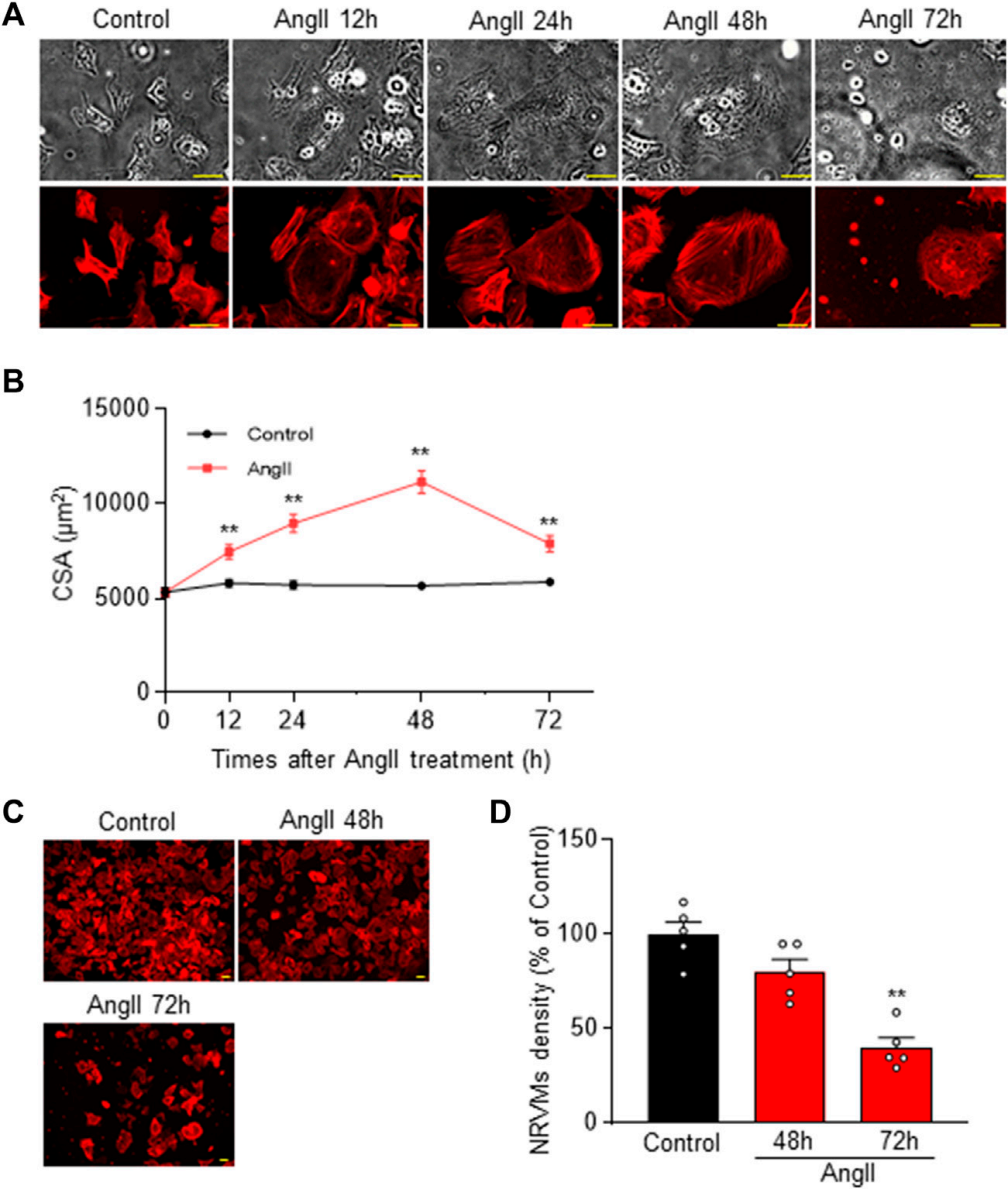
Cardiomyocyte hypertrophy in Neonatal Rat Ventricular Myocytes (NRVM) induced by 48 h after AngII challenge. **(A)** Time course of AngII-induced cardiomyocyte hypertrophy in NRVM. Representative cells are shown in transmitted light images (upper row) and rhodamine-conjugated phalloidin-stained fluorescence images (lower row). Hypertrophy of NRVM cells occurs up to 72 h (h) after administration of 100 nM AngII. Scale bar = 50 µm. **(B)** Quantification of CSA from the rhodamine-conjugated phalloidin stained images in **(A)**, demonstrating the temporal progression of CSA across multiple samples (*n* = 102–112). **(C)** Rhodamine-conjugated phalloidin staining shows cell density at 48 and 72 h post AngII administration. Scale bar = 50 µm. **(D)** Statistical analysis of cell density based on multiple observations as depicted in **(C)** (*n* = 5). Each column represents the mean ± SEM. **, *p* < 0.01 versus control cells.

As shown in Figure 1, we observed cardiomyocyte hypertrophy at 0, 12, 24, and 48 h after 100 nM AngII administration. The CSA increased to 140.09% ± 6.90%, 168.43% ± 8.85%, and 209.46% ± 11.51% (*n* = 102–112), respectively. These findings are consistent with previous reports on cell hypertrophy induced by AngII (Feng et al., 2019). Notably, the most significant cardiomyocyte hypertrophy was observed 48 h after AngII administration, and cytotoxicity became apparent at 72 h. Based on these observations, we decided to conduct further experiments at the 48 h.

Building upon the positive outcomes observed in previous cases and reports regarding the efficacy of Japanese Kampo medicines in cardiac amyloidosis, myocarditis, and HF [TJ-20 (Gautam et al., 2014), TJ-27 (Shijie et al., 2010), TJ-36 (Miho et al., 2019), TJ-126 (Imamura et al., 2022)], our study focused on investigating their direct effects on ventricular myocytes. This investigation aimed to assess the impact of these medicines. In addition, to explore unexplored aspects, we included TJ-19 (Amagaya et al., 2001) and TJ-54 (Yoshida et al., 2022) demonstrating an exacerbating effect on electrocardiograms and conducted a comprehensive analysis involving six specific herbal medicines.

Previous reports of cell-based Kampo medicines screening tests have shown efficacy at concentration ranging from 250 to 500 μg/mL (Liao et al., 2013; Teklemichael et al., 2020). In line with these reports, we examined the effect of these herbal medicines on AngII-induced cardiomyocyte hypertrophy at a concentration of 500 μg/mL.

**FIGURE 2:**
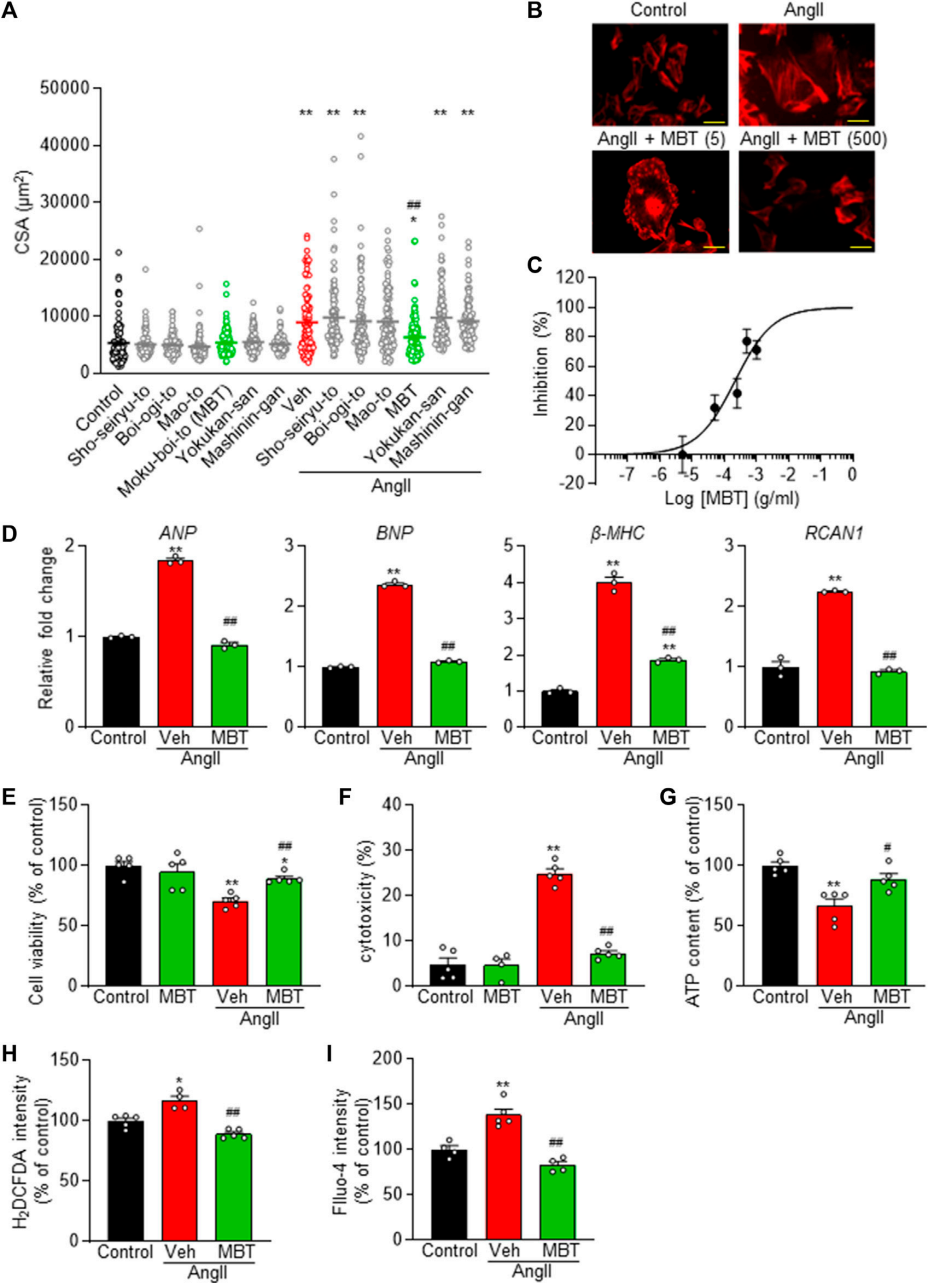
Dose-dependent prevention of AngII-induced cardiomyocyte hypertrophy and preservation of cell viability in NRVM by Japanese Kampo Moku-Boi-To. **(A)** Evaluation of the antihypertrophic effect of six types of Japanese Kampo medicines on AngII-induced myocardial hypertrophy. Each group comprises more than 100 cells (*n* = 101–118). **(B)** Dose-dependent inhibition of 100 nM AngII-induced hypertrophy by MBT. Representative image of cells stained with rhodamine-conjugated phalloidin at each concentration. Scale bar = 50 µm. **(C)** Statistical analysis of dose-dependent inhibition curve of MBT against 100 nM AngII-induced hypertrophy, based on multiple observations as depicted in **(B)**. Each group comprises more than 100 cells (*n* = 100–111). **(D–I)** Evaluation of the effects of AngII and MBT. NRVMs were treated with control, 100 nM AngII treatment (Veh: vehicle), or AngII +500 μg/mL MBT. **(D)** Quantitative real-time PCR analysis of hypertrophic gene markers, including *ANP*, *BNP*, *β-MHC*, and *RCAN1*. **(E)** Cell viability (*n* = 5). **(F)** Cytotoxicity (*n* = 4–5). **(G)** Intracellular ATP content (*n* = 4–5). **(H)** Intracellular ROS generation (H_2_DCFDA intensity) (*n* = 4–5). **(I)** Intracellular Ca^2+^ concentration (Fluo-4 intensity) (*n* = 4–5). Each column represents the mean ± SEM. *, *p* < 0.05; **, *p* < 0.01 indicate a significant difference compared to control cells. #, *p* < 0.05, ##, *p* < 0.01 indicate a significant difference compared to AngII + vehicle (Veh) treated cells.

Six herbal medicines treatment in control NRVMs had no effect on CSA. However, administration of MBT (Hypertrophic cells + MBT) partially but significantly inhibited cell hypertrophy at 48 h after AngII administration (Figures 2A, B), whereas none of the other five Japanese Kampo medicines (Sho-seiryu-to, Boi-ogi-to, Mao-to, Yokukan-san, and Mashinin-gan) showed any significant effect. Subsequently, we investigated the dose dependence of MBT on AngII-induced hypertrophy. MBT dose-dependently inhibited hypertrophy induced by AngII, with an IC_50_ of 214.7 μg/mL and Hill slope of 0.7 (Figure 2C). Based on these results, we treated the cells with MBT at a 500 μg/mL concentration to further investigate the factors associated with the anti-hypertrophic effect, specifically at 48 h following AngII treatment.

To further elucidate the effect of MBT on cardiac hypertrophy, we conducted qRT-PCR experiments to measure the expression of *ANP*, *BNP*, *β-MHC*, and *RCAN1* mRNA levels, all of which serve as markers of cardiac hypertrophy (Figure 2D). In response to AngII treatment, the expression of these markers increased significantly, but their levels were suppressed by MBT treatment. Additionally, MBT ameliorated AngII-induced cellular cytotoxicity (Figures 2E, F). Importantly, treatment with MBT alone did not result in any significant changes compared to control cells, demonstrating that MBT at a concentration of 500 μg/mL does not induce cardiomyocyte toxicity (Figures 2E, F).

Cardiomyocyte hypertrophy is known to be associated with abnormalities in elevated intracellular levels of ATP, ROS, and [Ca^2+^]_i_ (Brown et al., 2017; Quiles and Gustafsson Å, 2022). To investigate the protective effect of MBT, we investigated intracellular signals when NRVM was treated with AngII for 48 h. The results depicted in Figures 2G–I reveal that the AngII-treated group exhibited a notable reduction in ATP content and increase in intracellular ROS and Ca^2+^ concentrations. These results are consistent with previous reports (Wang et al., 2012; Tagashira et al., 2014; Zhao et al., 2020) regarding the effects of AngII on ATP, ROS, and [Ca^2+^]_i_ levels. However, in the MBT-treated group, the AngII-induced depletion of ATP and elevation in ROS and [Ca^2+^]_i_ were effectively mitigated, indicating the protective effect of MBT against these abnormalities.

Collectively, these results provide strong evidence that MBT effectively mitigates AngII-induced cardiomyocyte hypertrophy and cell death.

### Effects of MBT on mitochondrial morphology and function in AngII-treated NRVMs

**FIGURE 3:**
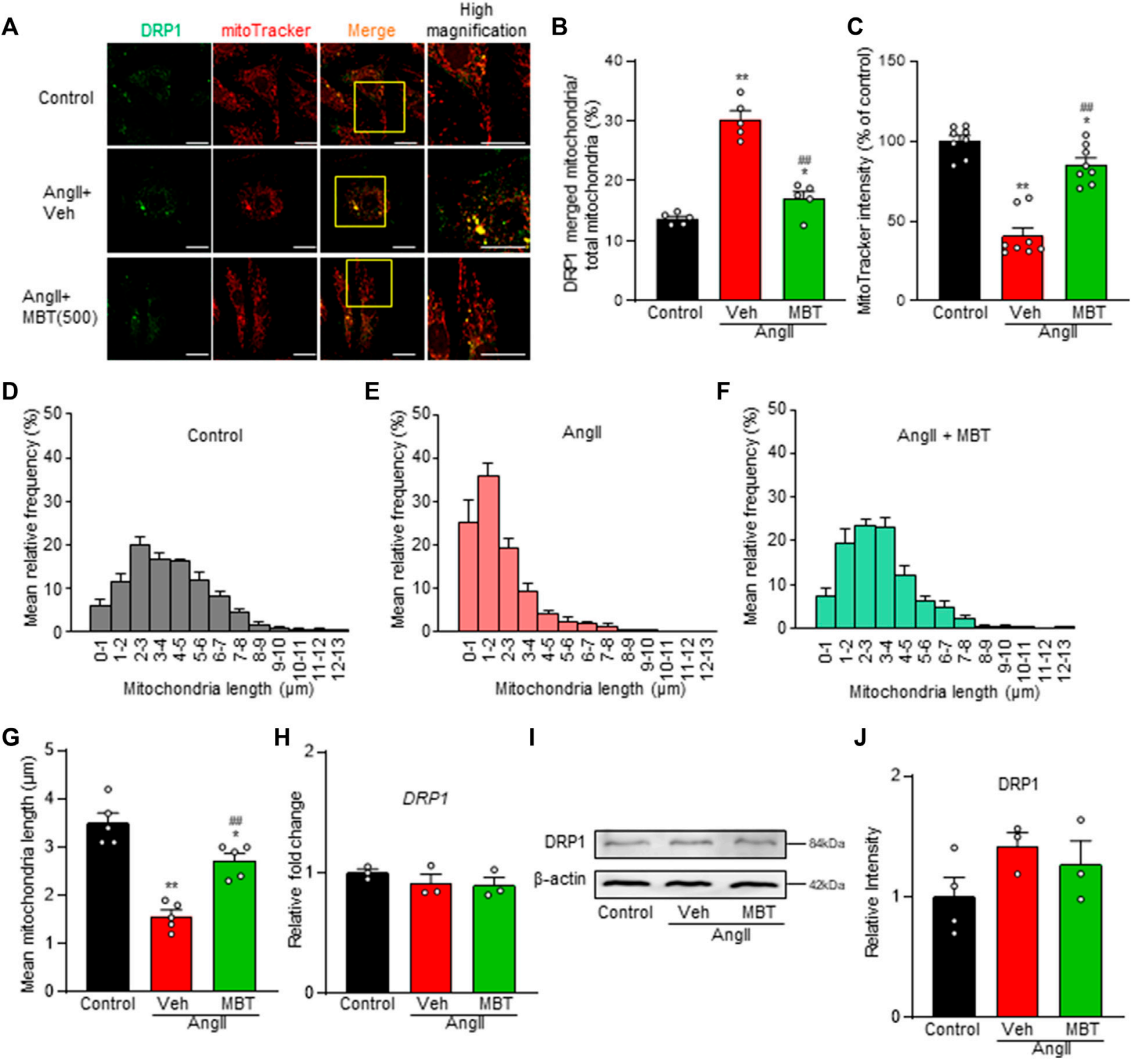
Mitochondrial Improvement by MBT in AngII-Treated NRVMs. Mitochondrial assessment of NRVMs under different conditions: control, 100 nM AngII treatment [vehicle (Veh)], and AngII +500 μg/mL MBT. **(A)** Immunofluorescence imaging showing DRP1 (green) and mitochondria (red). Scale bar = 20 μm. **(B)** Ratio of DRP1-bound mitochondria to total mitochondria (*n* = 5). **(C)** Quantification of mitochondrial membrane potential using MitoTracker Red CMXRos intensity (*n* = 8–9). **(D–F)** Distribution of mitochondrial length (*n* = 5). **(G)** Bar graph representing the average mitochondrial length obtained by Gaussian fitting of data from **(D–F)** (*n* = 5). **(H)** Quantitative real-time PCR analysis of DRP1 (*n* = 3). **(I)** Representative images of Western blot analysis of DRP1 and β-actin expression are shown. **(J)** Densitometric quantification of DRP1/β-actin immunoreactive bands (*n* = 3–4). Each column represents the mean ± SEM. *, *p* < 0.05 and **, *p* < 0.01 indicate significant differences compared to the control. ##, *p* < 0.01 indicates a significant difference compared to AngII + vehicle (Veh) treated cells.

Figures 3A, B demonstrates increased mitochondrial accumulation of DRP1, a protein essential for mitochondrial fragmentation, in the AngII-treated group (AngII). Additionally, a decrease in the fluorescence intensity of MitoTracker Red CMXRos, a marker that enters mitochondria in a voltage-dependent manner, was observed (Figures 3A, C, AngII, Veh). Mitochondrial fragmentation was enhanced, leading to a significant decrease in mitochondrial length compared to control NRVMs, although the expression level of DRP1 was not affected (Figures 3A, D, E, G–J). These findings indicate that AngII treatment induces abnormalities in mitochondrial morphology and function at 48 h, contributing to cardiomyocyte hypertrophy and death. In contrast, MBT treatment prevented the increased accumulation of DRP1 in mitochondria induced by AngII, restored the fluorescence intensity of MitoTracker Red CMXRos, and attenuated mitochondrial fragmentation (Figures 3A–C, F, G). Collectively, these results demonstrate that MBT directly acts on cardiomyocytes to ameliorate mitochondrial morphological and functional abnormalities.

### Effects of MBT on AngII receptors pathway in NRVMs

**FIGURE 4:**
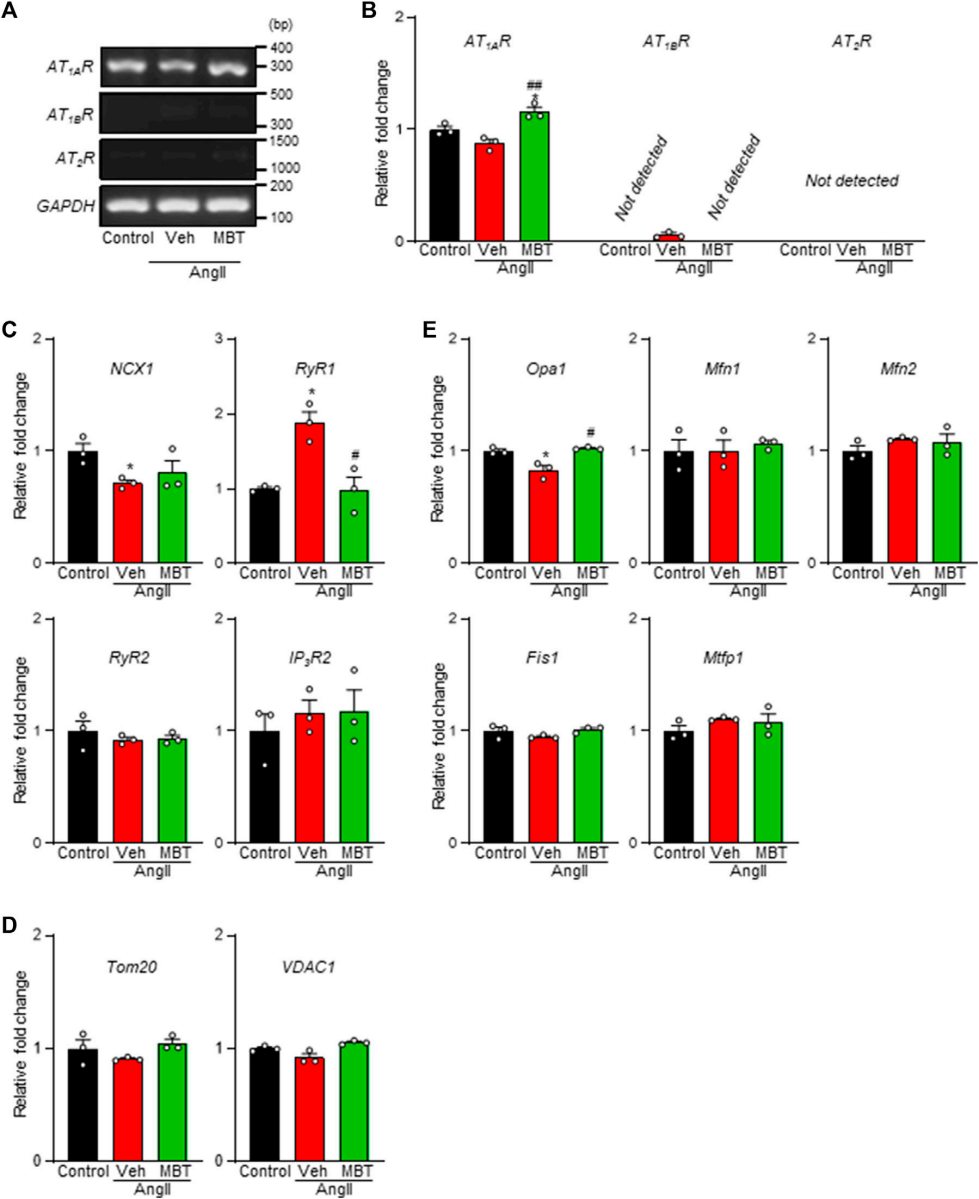
Expression of ATRs, Ca^2+^ homeostasis-related genes, and mitochondria-related genes in NRVMs. Evaluation of mRNA expression changes in ATRs, Ca^2+^ homeostasis-related genes, and mitochondrial fusion/fission factors in NRVMs under different conditions: control, 100 nM AngII treatment [vehicle (Veh)], and AngII +500 μg/mL MBT. **(A)** mRNA expression of ATRs. PCR products obtained from NRVMs treated under different conditions show the expression of *AT*_*1A*_ *R*, *AT*_*1B*_ *R*, *AT*_*2*_ *R*, and a constitutively transcribed control *GAPDH*. Nucleotide sequences of the PCR products obtained using ATRs-specific primers are identical to the corresponding sequences of *AT*_*1A*_ *R* (306 bp), *AT*_*1B*_ *R* (345 bp), *AT*_*2*_ *R* (1126 bp), and *GAPDH* (140 bp), respectively. **(B)** Quantitative real-time PCR analysis of *AT*_*1A*_ *R*, *AT*_*1B*_ *R*, and *AT*_*2*_ *R* mRNA levels (*n* = 3). **(C)** Quantitative real-time PCR analysis of Ca^2+^ homeostasis-related genes *NCX1*, *RyR1*, *RyR2*, and *IP*_*3*_ *R2* (*n* = 3). **(D)** Quantitative real-time PCR analysis of mitochondrial transport-related factors *Tom20* and *VDAC1* (*n* = 3). **(E)** Quantitative real-time PCR analysis of mitochondrial fusion factors *Mfn1/2* and *Opa1* and mitochondrial fragmentation factors *Fis1* and *Mtfp1* (*n* = 3). Data are presented as fold changes compared to control NRVM values. Each column represents mean ± SEM. *, *p* < 0.05 indicates a significant difference compared to control. #, *p* < 0.05 and ##, *p* < 0.01 indicate significant differences compared to AngII + vehicle (Veh) treated cells.

Ang II receptor antagonists have been implicated in mitochondria protection and the modulation of Ca^2+^ signaling through the AT receptors (ATRs) pathway (Javadov and Escobales, 2015). Thus, we aimed to elucidate the expression patterns of the specific AngII receptor subtypes, AT_1_ and AT_2_, in NRVMs. Our analysis revealed a prominent expression of *AT*_*1A*_ *R* in NRVMs, while *AT*_*1B*_ *R* and *AT*_*2*_ *R* expression levels were minimal (Figures 4A, B). Although no significant changes were observed in AngII-treated NRVMs compared to control NRVMs, a notable declining trend in *AT*_*1A*_ *R* mRNA levels were evident (Figures 4A, B). On the other hand, *AT*_*1A*_ *R* mRNA expression was upregulated in the MBT-treated group (Figures 4A, B).

In the context of hypertrophic myocardium induced by AngII, intracellular Ca^2+^ levels are known to be elevated (Wang et al., 2012), and we observed an improvement in Ca^2+^ homeostasis in the MBT treatment group (Figure 2I). To further explore these findings, we conducted a qRT-PCR experiment to assess the expression of critical genes associated with Ca^2+^ homeostasis, specifically *IP*_*3*_ *R2*, *RyR1*, *RyR2*, and *NCX1* (Figure 4C). After 48 h of AngII treatment, a noticeable decrease in *NCX1* expression and concomitant increase in *RyR1* mRNA levels were observed. These changes indicated disturbances in Ca^2+^ homeostasis resulting from altered expression of these channels. Remarkably, MBT treatment mitigated the increase in RyR1 expression (Figure 4C), suggesting a potential role in restoring Ca^2+^ homeostasis.

Furthermore, considering the observed improvements in ATP and ROS levels in hypertrophic myocardium following MBT treatment (Figures 2G, H), we examined the mRNA expression levels of mitochondrial proteins associated with membrane permeability and chloride channels, including *Tom20* and *VDAC1* (Chacinska et al., 2009; Camara et al., 2017). However, the expression of these mitochondrial-related genes remained unchanged upon stimulation with AngII or administration of MBT treatment (Figure 4D). Subsequently, we explored the mRNA expression of genes related to mitochondrial fusion (*Mfn1/2* and *Opa1*) and mitochondrial fragmentation (*Fis1* and *Mtfp1*) (Donnarumma et al., 2022; Wu et al., 2023) (Figure 4E). Notably, *Opa1*, a mitochondrial fusion protein, exhibited a significant reduction in response to AngII treatment, indicative of mitochondrial fragmentation. In contrast, MBT treatment effectively attenuated this decrease in *Opa1* expression induced by AngII. On the other hand, the mRNA expression levels of *Mfn1/2*, *Fis1*, and *Mtfp1* did not change with AngII stimulation or MBT treatment. These results collectively suggest that MBT administration may play a role in ameliorating Ca^2+^ signaling abnormalities and mitochondrial fragmentation induced by AngII.

Combination effects of MBT and losartan on AngII-induced cardiomyocyte hypertrophy and death in cultured NRVMs.

Based on the results of AT_1_Rs expression in NRVMs, we explored the possibility that AT_1_R-mediated AngII affects cardiomyocyte hypertrophy. To examine the additional effect of MBT on AngII-induced cardiac hypertrophy, we used the AT_1_R inhibitor losartan.

**FIGURE 5:**
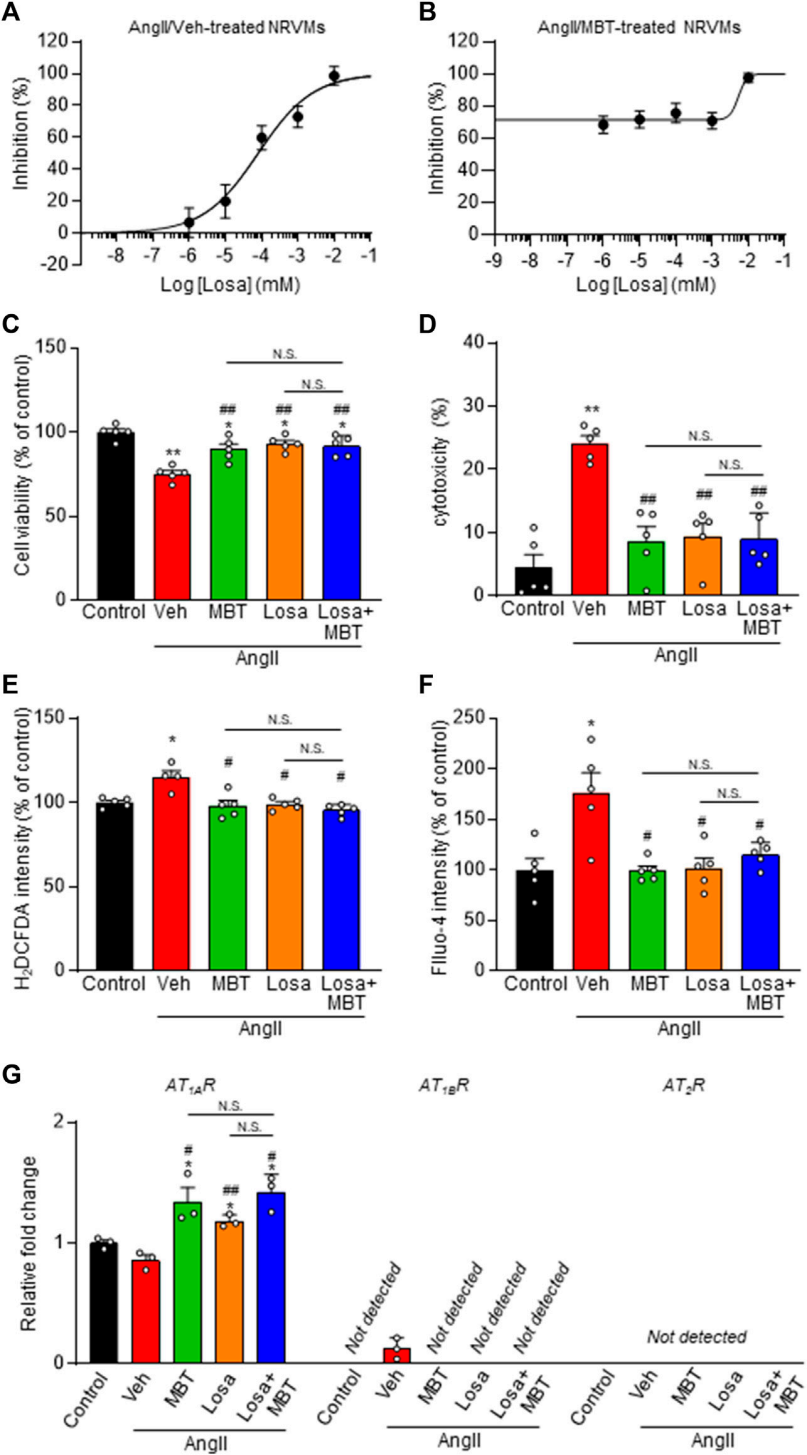
Effect of losartan and MBT on NRVMs. **(A,B)** Evaluation of NRVM CSA based on rhodamine-conjugated phalloidin staining in response to 100 nM AngII-induced hypertrophy. **(A)** Dose-dependent inhibition curve of losartan (Losa) (*n* = 101–111). **(B)** Dose-dependent inhibition curve of losartan co-treated with AngII and 500 μg/mL MBT (*n* = 104–115). Each group consists of more than 100 cells. **(C–F)** Evaluation of the effects of control, vehicle (Veh), 500 μg/mL MBT, 1 μM Losa, or co-treatment of 1 μM losartan and 500 μg/mL MBT on 100 nM AngII-induced cardiomyocyte hypertrophy, including cell viability (*n* = 5) **(C)**, cytotoxicity (*n* = 5) **(D)**, ROS production (H_2_DCFDA intensity) (*n* = 4–5) **(E)**, and intracellular Ca^2+^ concentration (Fluo-4 intensity) (*n* = 5) **(F)**. **(G)** Quantitative real-time PCR analysis of ATRs (*n* = 3). Each column represents mean ± SEM. *, *p* < 0.05; **, *p* < 0.01 indicate significant differences compared to control cells. #, *p* < 0.05; ##, *p* < 0.01 indicate significant differences compared to AngII + vehicle (Veh) treated cells. N.S. indicates no significant difference.

AngII-induced cardiomyocyte hypertrophy was suppressed by losartan, with an IC_50_ of 80.0 nM and Hill slope of 0.6 (Figure 5A). Notably, the concentration-dependent inhibition curve of losartan in the presence of 500 μg/mL MBT showed a substantial effect at losartan concentrations equal to or greater than 1 μM (Figure 5B). Moreover, losartan effectively counteracted the decrease in cell viability, and the increase in cytotoxicity, ROS production, and the increase in [Ca^2+^]_i_ induced by AngII (Figures 5C–F). Intriguingly, when comparing the combination treatment of losartan and MBT with the individual therapies, no significant difference were observed in their inhibitory effects on these AngII-induced responses (Figures 5C–F).

**FIGURE 6:**
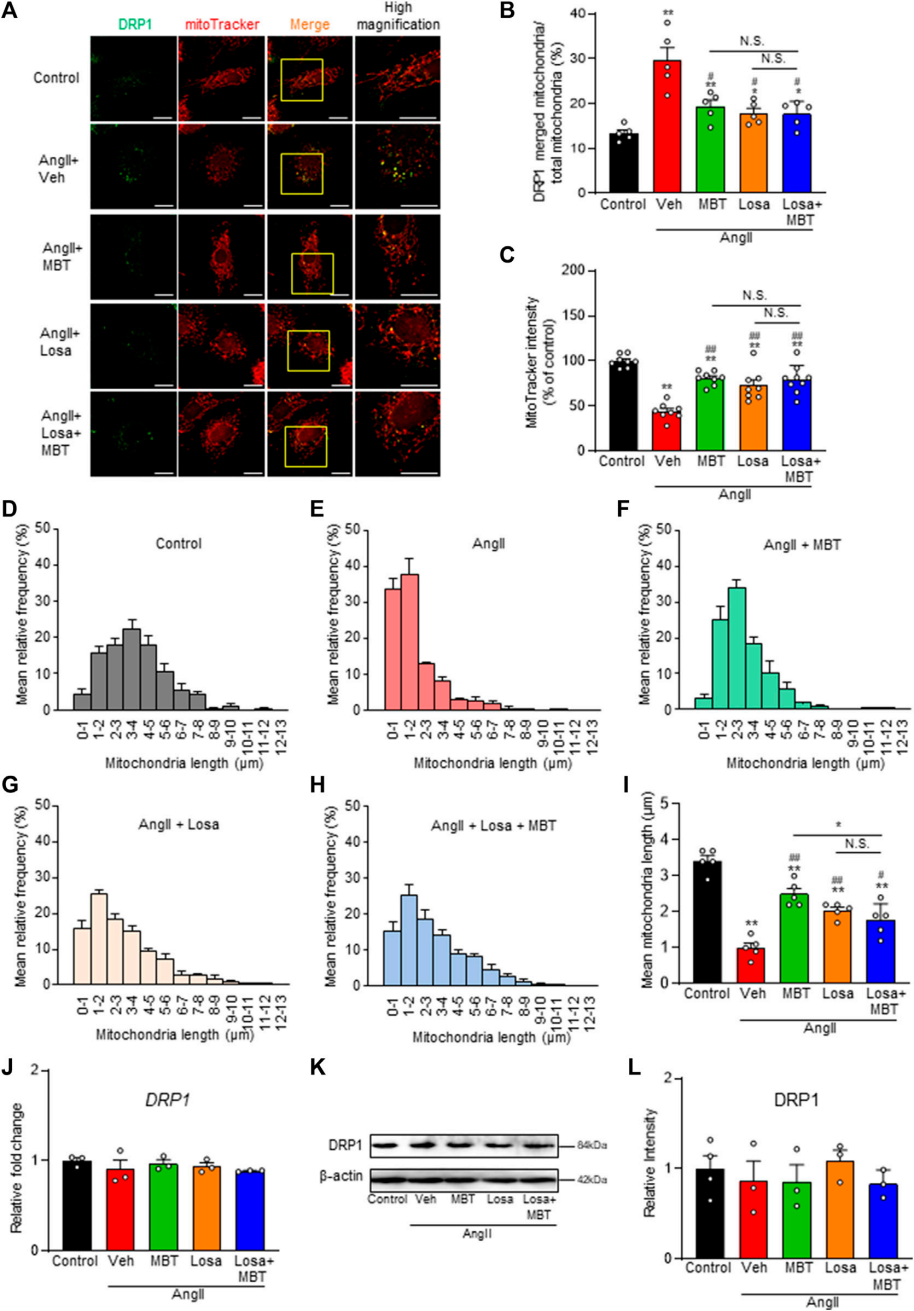
Effect of losartan and MBT co-administration on mitochondrial morphology and dysfunction in AngII-induced myocardial hypertrophy. Evaluation of mitochondrial changes induced by 100 nM AngII, control, vehicle (Veh), 500 μg/mL MBT, 1 μM losartan (Losa), or co-treatment of 1 μM losartan and 500 μg/mL MBT (Losa + MBT). **(A)** Immunofluorescence image showing DRP1 in green and mitochondria in red. Scale bar = 20 μm. **(B)** Ratio of DRP1-bound mitochondria/total mitochondria (*n* = 5). **(C)** Quantification of mitochondrial membrane potential using MitoTracker Red CMXRos intensity (*n* = 8–9). **(D–H)** Distribution of mitochondrial length measured from images in A (*n* = 5). **(I)** Bar graph representing the average mitochondrial length obtained by Gaussian fitting from individual panels **(D–H)** (*n* = 5). **(J)** Quantitative real-time PCR analysis of DRP1 (*n* = 3). **(K)** Representative images of Western blot analysis showing DRP1 and β-actin expression. **(L)** Densitometric quantification of DRP1/β-actin immunoreactive bands (*n* = 3–4). Each column represents the mean ± SEM. *, *p* < 0.05 and **, *p* < 0.01, indicating a significant difference compared to control cells. #, *p* < 0.05 and ##, *p* < 0.01 denote a significant difference compared to AngII + vehicle (Veh) treated cells. N.S. indicates no significant difference.

Additionally, we investigated the mRNA levels of ATRs. Our findings indicate that both losartan and MBT treatments, whether administered individually or in combination, increased *AT*_*1A*_ *R* expression (Figure 5G). These results suggest that similar to losartan, MBT may function by blocking *AT*_*1A*_ *R*, leading to its upregulation. Subsequently, we explored the impact of losartan and MBT, either alone or in combination, on mitochondrial morphology and function during AngII-induced cardiomyocyte hypertrophy. We assessed mitochondrial damage by evaluating DRP1 accumulation, function, and morphology. Interestingly, no significant differences were observed in the reduction of DRP1 aggregation upon AngII stimulation when comparing the losartan or MBT monotherapy group to the combination treatment group (Figures 6A, B).

Additionally, we measured a decrease in the fluorescence intensity of MitoTracker Red CMXRos, and both losartan and MBT monotherapy effectively ameliorated this decrease (Figures 6A, C). However, the combination of losartan and MBT did not provide any additional effect. Furthermore, we observed that AngII-induced mitochondrial fragmentation was ameliorated by losartan or MBT alone, but co-administration of losartan and MBT exerted no further effects (Figures 6A, D–H). Notably, there was no change in DRP1 expression among the different treatment groups (Figures 6J–L).

### Effect of MBT on isoproterenol-induced cardiac hypertrophy and dysfunctions in mice

**FIGURE 7:**
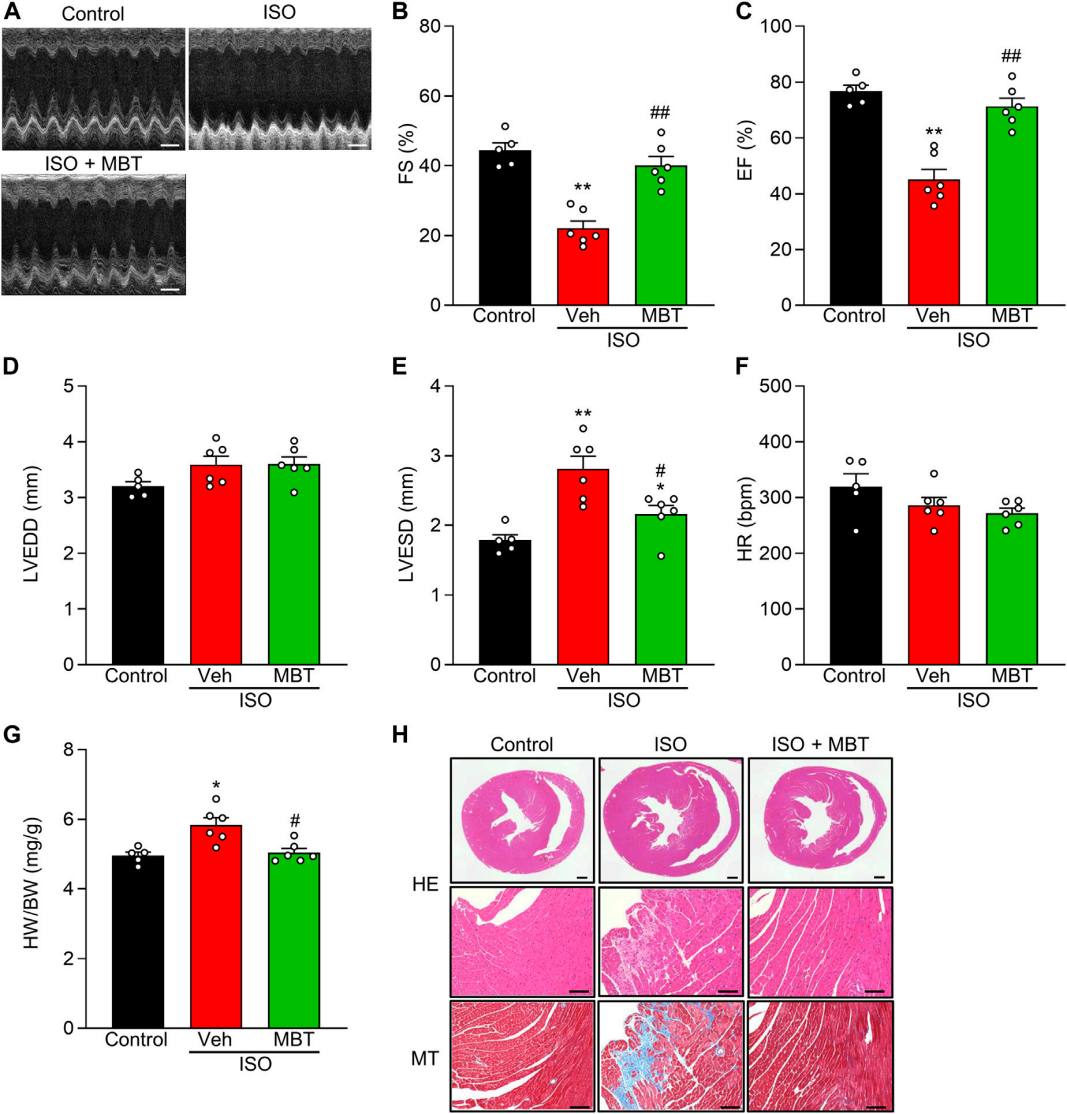
Effect of MBT on ISO-induced cardiac hypertrophy and dysfunctions *in vivo*. **(A)** Representative M-mode echocardiograms of mice with or without ISO and/or MBT treatments. Scale bar = 200 ms. **(B)** Changes in percentage of left ventricular (LV) fractional shortening (FS%) (*n* = 5–6). **(C)** Changes in percentage of LV ejection fraction (EF%) (*n* = 5–6). **(D)** Changes of left ventricular LV end-diastolic diameter (LVEDD) (*n* = 5–6). **(E)** Changes of LV end-systolic diameter (LVESD) (*n* = 5–6). **(F)** Changes in Heart rate (HR) (*n* = 5–6). **(G)** ISO-induced cardiac hypertrophy as indicated by the heart weight/body weight (HW/BW) ratio (*n* = 5–6). **(H)** Profiles of heart tissues in mice treated with or without ISO and/or MBT (HE staining and Masson staining) (*n* = 5–6). Scale bar = 500 µm (whole heart) and 100 µm (High magnification), respectively. Each column represents the mean ± SEM. *, *p* < 0.05 and **, *p* < 0.01, indicating a significant difference compared to control mice. #, *p* < 0.05 and ##, *p* < 0.01 denote a significant difference compared to ISO + vehicle (Veh) treated mice.

To assess the protective effect of MBT against ISO-induced cardiac hypertrophy and dysfunction model, we conducted echocardiography in mice (Figure 7A). MBT was administered at a concentration of 500 mg/kg based on previous *in vivo* studies (Wang et al., 1998) and our NRVM findings in this study.

ISO treatment (30 mg/kg body weight, once a day, i.p.) significantly reduced LV FS% and EF% while concurrently increasing LVESD and HW/BW compared to control mice (Figures 7B–E, G). These findings were consistent with previous reports (Park et al., 2018; Cheng et al., 2020). Notably, the administration of MBT (500 mg/kg body weight, p.o.) ameliorated ISO-induced cardiac hypertrophy and cardiac dysfunction (Figures 7B–E, G). HR measurements were performed to ensure that anesthesia depth did not interfere with the results. No significant changes were observed in any of the experimental groups (Figure 7F). Histological examination of heart tissue from the model group revealed prominent tissue fibrosis and collagen deposition. At the same time, MBT administration prevented the occurrence of these injuries (Figure 7H). In conclusion, our results suggest that MBT effectively improves ISO-induced cardiac hypertrophy and cardiac dysfunction in *in vivo* model mice.

## Discussion

The effects of Japanese Kampo medicines on the heart, particularly cardiac mitochondrial function and Ca^2+^ signaling, remain poorly understood despite their widespread use for various diseases. In this study, we conducted a screening analysis and demonstrated, for the first time, that MBT directly acts on cardiomyocytes to prevent AngII-induced hypertrophy and cell death. Furthermore, we found that MBT protects against AngII-induced disruption of intracellular Ca^2+^ homeostasis and mitochondrial dysfunction. In addition, MBT improves ISO-induced cardiac hypertrophy and cardiac dysfunction *in vivo*. Although the exact mechanism of action is not fully elucidated, our findings suggest that these effects may be mediated, at least in part, by angiotensin type I receptor (AT_1_R) blocking.

Previous studies have highlighted the association between cardiomyocyte hypertrophy and impaired mitochondrial metabolism (Rosca et al., 2013). For instance, inhibition of mitochondrial fission has been reported to protect the heart against I/R injury *in vivo* (Ong et al., 2010). Additionally, treatment of neonatal rat ventricular myocytes (NRVMs) with AngII induces mitochondrial fission and apoptosis, which can be prevented by the knockdown of DRP1 (Qi et al., 2018). Furthermore, mitochondrial fission has been implicated in endothelin-1-induced hypertrophy and cell death, as well as leptin-induced hypertrophy (Jong et al., 2019; Tagashira et al., 2023). These studies collectively emphasize the importance of mitochondrial quality control in cardiomyocyte hypertrophy and death induced by various stimuli.

Mitochondrial fragmentation leads to increased reactive oxygen species (ROS) production, promotes mitophagy, and decreases ATP production (Tagashira et al., 2013; Tagashira et al., 2014; Yang et al., 2021; Quiles and Gustafsson Å, 2022). ATP depletion and elevated ROS production contribute significantly to the pathogenesis of HF (Doenst et al., 2013; Peoples et al., 2019). Consistent with these findings, our study revealed that AngII treatment induced mitochondrial fragmentation, while MBT treatment ameliorated this effect and suppressed the subsequent increase in ROS production and decrease ATP production (Figures 2G, H).

DRP1, an essential protein involved in mitochondrial fragmentation (Smirnova et al., 2001), accumulates in cardiac mitochondria, implying mitochondrial dysfunction (Aung et al., 2021). Mechanisms such as phosphorylation, mediated by norepinephrine-induced intracellular Ca^2+^ increase and calcineurin activation, have been reported (Pennanen et al., 2014; Aung et al., 2021). Mitochondrial fission 1 protein (Fis1) is another molecule that cooperates with DRP1 to contribute to mitochondrial fragmentation (Zerihun et al., 2023). Meanwhile, mitochondrial fission process protein 1 (Mtfp1) is a key player in mitochondrial fragmentation in cardiac muscle and play an essential role in maintaining heart structure and function (Tondera et al., 2005; Aung et al., 2017). In our study, we observed accumulation of DRP1 in mitochondria of AngII-treated NRVMs, which was ameliorated by MBT treatment, although the expression levels of *DRP1*, *Fis1*, and *Mtfp1* were not affected (Figures 3H–J, 4E).

Interestingly, *Opa1* mRNA exhibited a significant reduction in response to AngII, while MBT treatment attenuated this decrease in *Opa1* mRNA expression (Figure 4E). A previous study using H9c2 cells reported a similar reduction in *Opa1* mRNA with AngII treatment, while *DRP1* and *Fis1* mRNA levels remained unchanged (Gul et al., 2023), which aligns with the findings of our study. These results suggest that the regulation of *Opa1* expression may also play a partial role in the cardioprotective mechanism of MBT. Additionally, MBT inhibited the AngII-induced increase in [Ca^2+^]_i_ (Figure 2I), which might contribute to the inhibition of DRP1 accumulation by MBT.

Interestingly, MBT treatment alone resulted in nearly complete improvement in mitochondrial length distribution, suggesting its superiority in terms of mitochondrial quality control (Figures 3C, F, G). This indicates that the effects of MBT may extend beyond AT_1_R blocking. Furthermore, while MBT partially suppressed mitochondrial quality control and myocardial hypertrophy, it suppressed AngII-induced intracellular Ca^2+^ overload (Figures 2I, 5F). This improvement in Ca^2+^ homeostasis may be related to the regulation of RyR1 expression (Figure 4C). These results indicate that MBT may act on Ca^2+^-independent mitochondrial quality control mechanisms crucial for HF development. Detailed investigations are required to determine the precise mechanism by which MBT prevents AngII-induced mitochondrial dysfunction.

Among the six commonly used Japanese Kampo medicines that indicated cardiac effects in human and rodents investigated in this study, MBT was the only one that demonstrated inhibition of cardiac hypertrophy induced by AngII (Figure 2). Unexpectedly, neither inhibitory nor exacerbating effects on AngII-induced cardiomyocyte hypertrophy were detected with treatment of Sho-seiryu-to (TJ-19), Boi-ogi-to (TJ-20), Mao-to (TJ-27), and Yokukan-san (TJ-54), suggesting that the effect of treatment on humans and rodents is not a direct effect on cardiomyocytes or they lack anti-hypertrophic effects. On the other hand, mashinin-gan (TJ-126) had no adverse effects on NRVMs, consistent with previous report (Imamura et al., 2022), indicating that its safe use in patients with cardiac amyloidosis. MBT exhibited significant suppressive effects against AngII-induced hypertrophy, consistent with previous clinical research (Miho et al., 2019). Furthermore, we demonstrated that MBT mitigates cardiac dysfunction and cardiac hypertrophy accompanied by fibrosis in ISO-induced cardiac hypertrophy model mice (Figure 7). This finding aligns with a previous report, which indicated that MBT had a survival benefit in HF model mice (Wang et al., 1998).

Comprising four herbal medicines, MBT has traditionally been employed to alleviate dyspnea, wheezing, and cardiac edema accompanying HF. Although rare, hypersensitivity and gastrointestinal diseases have been reported as side effects. Overall, MBT is considered a safe therapeutic drug. In Japan, it has been utilized for the treatment of HF, and several case reports have highlighted its efficacy (Ezaki et al., 2019; Miho et al., 2019). For example, combination therapy with MBT and standard medical treatment was found to be effective in inoperable patients with severe aortic regurgitation (Miho et al., 2019). Furthermore, a randomized controlled trial demonstrated that MBT significantly improved HF symptoms in hospitalized patients with acute decompensated HF compared to standard care (Ezaki et al., 2019). Despite its clinical use, the direct effect of MBT on cardiomyocytes and its cardioprotective mechanism has yet to be fully elucidated. This study demonstrated that MBT protects against AngII-induced disruption of intracellular Ca^2+^ homeostasis and mitochondrial dysfunction (Figures 2, 3).

Losartan, a selective AT_1_R antagonist without affinity for AT_2_R (Schorb et al., 1993), has shown similar effects to angiotensin-converting enzyme inhibitors in animal experiments (Milavetz et al., 1996). Furthermore, losartan has been reported to have beneficial effects on cardiomyocyte contractility, Ca^2+^ regulation, post-infarction remodeling, and gene expression in rat models of chronic HF (Loennechen et al., 2002). Hence, the inhibition of AT_1_R is valuable for HF treatment.

In our study, we delved into the competitive inhibition of AT_1_R by investigating the combined effects of losartan and MBT in effectively treating cardiomyocyte hypertrophy and cell death. Specifically, we performed a detailed examination of the inhibitory effects of losartan in response to AngII stimulation in NRVM and investigated the potential synergy between losartan and MBT. Remarkably, the concentration-dependent inhibition curve of losartan in the presence of 500 μg/mL MBT showed substantial effects at losartan concentrations of 1 μM and higher (Figure 5B). The inhibition observed with 1 μM losartan in Figure 5B was 71.6%. Based on the data in Figures 2C, 5A, the concentrations of MBT and losartan required for 71.6% inhibition were determined to be 760.3 μg/mL and 409 nM, respectively. As a result, the combination index (CI) calculated for the combination of 1 μM losartan and 500 μg/mL MBT in Figure 5B was 3.1, suggesting the possibility of competitive inhibition between MBT and losartan on AT_1A_R. Notably, the deviation from the dose dependence of losartan alone observed at the 1 μM concentration in Figure 5B may be due to anomalous mole fraction effects resulting from the simultaneous binding of multiple substances to one binding site. It is worth mentioning that the phenomenon has been observed with the same multiple-occupancy theory in a related study (Lester and Dougherty, 1998).

Furthermore, in experiments aimed at confirming the effects of MBT on AngII stimulation, changes in cell viability, cytotoxicity, ROS production, ATP production, ATRs expression, and changes in mitochondrial function and morphology were thoroughly investigated (Figures 5, 6). The comprehensive findings showing that losartan and MBT exhibit similar behavior support the concept that MBT and losartan may target the same site and raise the possibility that AT_1A_R is potentially a target of MBT. These findings support the hypothesis that MBT may act on AT_1_R, although the precise nature of this interaction, whether direct or indirect, remains unclear.

Although it did not reach statistical significance, we observed a trend toward decreased *AT*_*1A*_ *R* expression in response to AngII. This decrease may be due to the increased ATR-mediated signal induced by AngII, leading to the downregulation of AT_1A_R to attenuate the signal strength. Interestingly, *AT*_*1A*_ *R* expression increased in response to AngII + MBT, AngII + losartan, and AngII + losartan + MBT treatments. This increase in *AT*_*1A*_ *R* expression is thought to be a compensatory mechanism to counteract the attenuation of AT_1A_R-mediated signals. This observation of such a compensatory mechanism is consistent with the previous finding that losartan can upregulate *AT*_*1*_ *R* in ischemia/reperfusion (I/R) rat hearts (Xu et al., 2002). These results suggest that similar to losartan, MBT may inhibit AT_1A_R, leading to its upregulation. These findings support the hypothesis that MBT exerts its effects by blocking AT_1A_R. However, in the case of *AT*_*1B*_ *R* or *AT*_*2*_ *R* expression, it remained consistently low, and the underlying mechanism of this phenomenon remains unknown.

In conclusion, our study demonstrates that MBT acts directly on cardiomyocytes, protecting against AngII-induced cardiomyocyte injury and ISO-induced cardiac dysfunction through the regulation of intracellular Ca^2+^ signaling and mitochondrial quality control. These effects may be mediated, at least in part, by the blocking of AT_1_R. The findings from this study support the potential use of MBT as an adjunctive therapy for HF. Further research is needed to fully elucidate the underlying mechanisms and explore the clinical implications of MBT treatment in HF management.

## Data Availability

The raw data supporting the conclusion of this article will be made available by the authors, without undue reservation.
